# Iodido{4-phenyl-1-[1-(1,3-thia­zol-2-yl-κ*N*)ethyl­idene]thio­semicarbazidato-κ^2^ *N*′,*S*}{4-phenyl-1-[1-(1,3-thia­zol-2-yl)ethyl­idene]thio­semicarbazide-κ*S*}cadmium(II)

**DOI:** 10.1107/S160053681300915X

**Published:** 2013-04-10

**Authors:** Ramaiyer Venkatraman, Dasary S. Samuel, Zikri Arslan, Md. Alamgir Hossain, Frank R. Fronczek

**Affiliations:** a Department of Chemistry and Biochemistry, Jackson State University, Jackson, MS 39217-0510, USA; b Department of Chemistry, Louisiana State University, Baton Rouge, LA 70803, USA

## Abstract

In the title complex, [Cd(C_12_H_11_N_4_S_2_)I(C_12_H_12_N_4_S_2_)], the Cd^II^ ion is penta­coordinated by two thio­semicarbazone ligands (one neutral and the other anionic) and one iodide ion in a distorted square pyramidal (τ = 0.35) geometry. The central ion is coordinated by the thia­zole N atom, the thio­ureido N and the S atom of the deprotonated thio­semicarbazone ligand. The other ligand is linked with the central ion through the C=S group. The deprotonated ligand intra­molecularly hydrogen bonds to the thia­zole ring N atom, while the ligand forms an inter­molecular hydrogen bond to the thiol­ate S atom of the second ligand. The deprotonation of the tridentate ligand and its coordination to the Cd^II^ ion *via* the S atom strikingly affects the C—S bond lengths. The C—S bond lengths in the neutral and deprotonated ligands in the metal complex are 1.709 (3) and 1.748 (2) Å, respectively, whereas it is 1.671 (3) Å in the free ligand. In the metal complex, the Cd—S distances are 2.6449 (6) and 2.5510 (6) Å. The Cd—I bond length is 2.7860 (2) Å.

## Related literature

For properties of thio­semicarbazones and Cd complexes, see: Casas *et al.* (2000 ▶); Milczarska *et al.* (1998 ▶); Venkatraman *et al.* (2009 ▶); Dasary *et al.* (2011 ▶); Viñuelas-Zahínos *et al.* (2011 ▶); Arumugam *et al.* (2011 ▶). For a description of the geometry of complexes with five-coordinate metal atoms, see: Addison *et al.* (1984 ▶). 
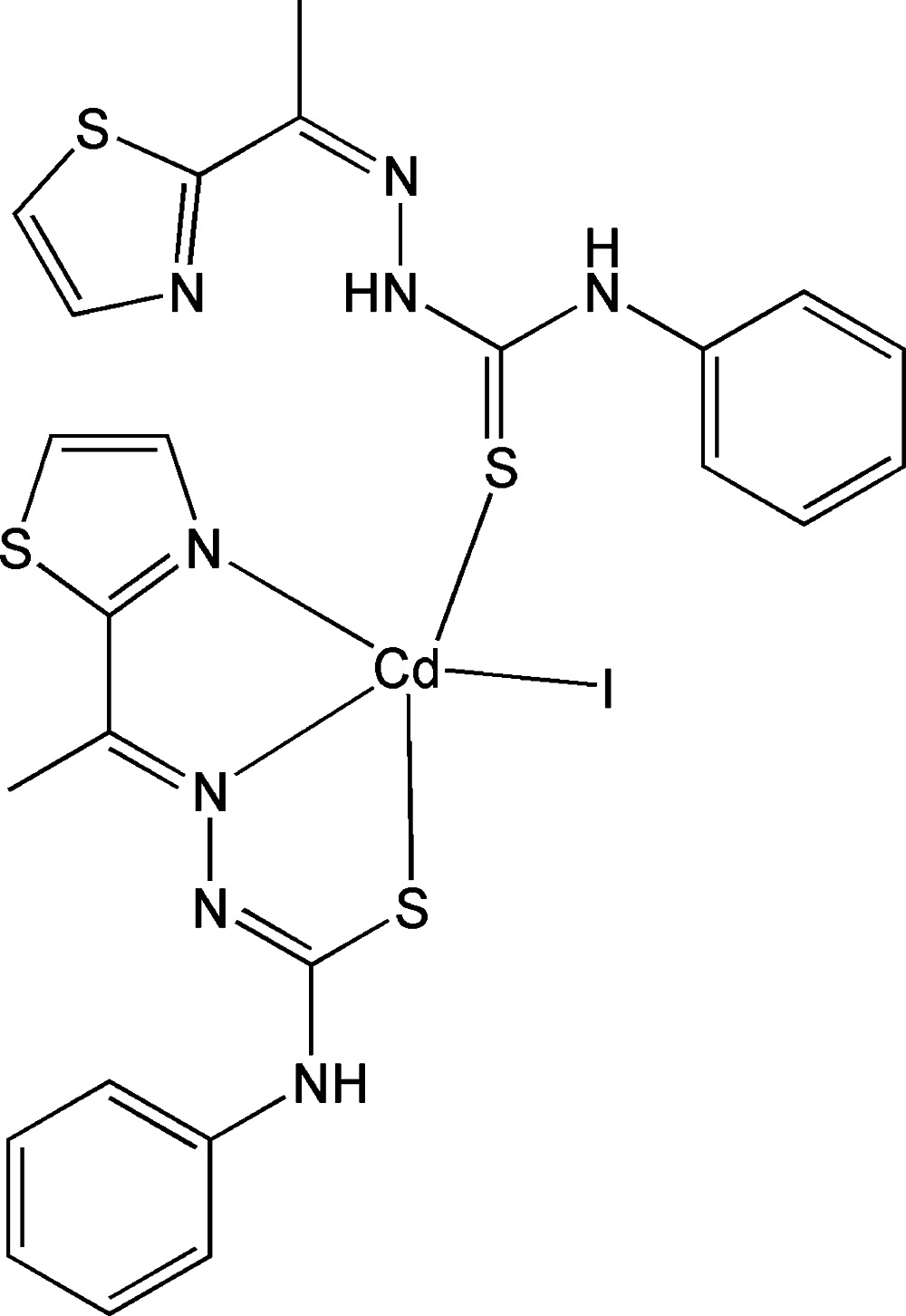


## Experimental

### 

#### Crystal data

[Cd(C_12_H_11_N_4_S_2_)I(C_12_H_12_N_4_S_2_)]
*M* *_r_* = 791.04
Triclinic, 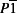

*a* = 8.6685 (4) Å
*b* = 10.1323 (5) Å
*c* = 16.7220 (8) Å
α = 76.607 (2)°
β = 79.481 (2)°
γ = 77.910 (2)°
*V* = 1383.15 (11) Å^3^
*Z* = 2
Mo *K*α radiation
μ = 2.24 mm^−1^
*T* = 90 K
0.13 × 0.06 × 0.05 mm

#### Data collection

Bruker Kappa APEXII DUO CCD diffractometer
Absorption correction: multi-scan (*SADABS*; Sheldrick, 2002 ▶) *T*_min_ = 0.760, *T*_max_ = 0.896
14913 measured reflections
8142 independent reflections
6715 reflections with *I* > 2σ(*I*)
*R*_int_ = 0.024

#### Refinement

*R*[*F* ^2^ > 2σ(*F* ^2^)] = 0.027
*wR*(*F* ^2^) = 0.060
*S* = 1.02
8142 reflections
354 parameters
3 restraints
H atoms treated by a mixture of independent and constrained refinement
Δρ_max_ = 0.92 e Å^−3^
Δρ_min_ = −0.60 e Å^−3^

### 

Data collection: *APEX2* (Bruker, 2005 ▶); cell refinement: *SAINT* (Bruker, 2002 ▶); data reduction: *SAINT*; program(s) used to solve structure: *SHELXS97* (Sheldrick, 2008 ▶); program(s) used to refine structure: *SHELXL97* (Sheldrick, 2008 ▶); molecular graphics: *ORTEP-3 for Windows* (Farrugia, 2012 ▶); software used to prepare material for publication: *SHELXL97*.

## Supplementary Material

Crystal structure: contains datablock(s) global, I. DOI: 10.1107/S160053681300915X/bv2219sup1.cif

Structure factors: contains datablock(s) I. DOI: 10.1107/S160053681300915X/bv2219Isup2.hkl

Additional supplementary materials:  crystallographic information; 3D view; checkCIF report

## Figures and Tables

**Table 1:** Hydrogen-bond geometry (Å, °)

*D*—H⋯*A*	*D*—H	H⋯*A*	*D*⋯*A*	*D*—H⋯*A*
N4—H4*N*⋯S2^i^	0.84 (2)	2.74 (2)	3.559 (2)	166 (2)
N7—H7*N*⋯N5	0.86 (2)	1.90 (2)	2.651 (3)	144 (3)

Symmetry code: (i) 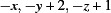.

**Fig. 1.**
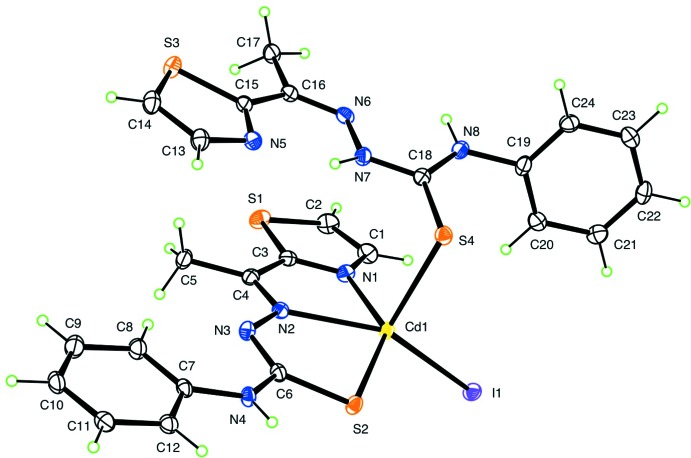
The molecular structure of the title compound. Displacement ellipsoids are drawn at the 50% probability level.

**Fig. 2.**
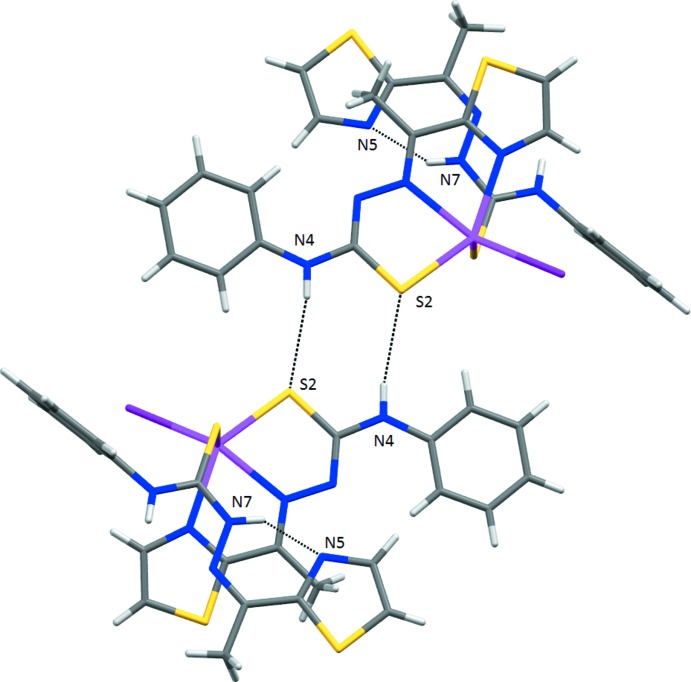
Intra- and inter-molecular H-hydrogen bonding of the compound (1) viewed along b axis.

## supplementary crystallographic information

### Comment

Heterocyclic thiosemicarbazones are versatile ligands forming complexes with a variety of transition metal ions. These ligands and their metal complexes are found to exhibit cytotoxic effects (Casas *et al.*, 2000, Milczarska *et al.*, 1998). Among several metals ions that complex with thiosemicarbazones, cadmium (II) has received less attention (Viñuelas-Zahínos *et al.* 2011). In continuation of our structural studies of metal thiosemicarbazones (Venkatraman *et al.*, 2009; Dasary *et al.*, 2011; Arumugam *et al.* 2011), we herein report the Cd^II^ complex of 2- acetyl thiazole N(4) phenyl thiosemicarbazone. The title complex is obtained from the reaction of cadmium (II) iodide with two equivalents of neutral ligands in methanol. This complex is an isomorph of the Hg complex reported by us earlier (Dasary *et al.*, 2011). In this complex, the Cd^II^ is tridentately attached to one of the deprotonated ligand through the donor groups of N1, N2 and S2, while the metal ion is singly coordinated to the other ligand *via* S4. As shown in Fig. 1, one iodide is found to coordinate with the central metal ion from other side forming a pentacoodinated complex (τ= 0.35) with a pyramidal square planar geometry (Addison *et al.*, 1984). The deprotonated ligand is twisted due to intra-molecularly hydrogen bonds (N7H···N5), while the ligand forms an intermolecular hydrogen bond *via* S2 with NH group (N4) from the other ligand (Fig. 2). The deprotonation of the tridentate ligand and its coordination to the Cd *via* the S atom strikingly affects the C—S bond lengths. The C—S bond distances in the neutral and deprotonated ligands in the metal complex are 1.709 (3) Å and 1.748 (2) Å respectively whereas it is 1.671 (3) Å in the free ligand. In the metal complex, the Cd—S distances are 2.6449 (6) Å, 2.5510 (6) Å. The Cd—I bond distance is 2.7860 (2) Å. The torsion angles (N2—N3—C6—N4), and (N6—N7—C18—N8) for two ligands is 179.48 (8)° and 8.5 (3)° respectively. Hydrogen bonding details are given in Table 1.

### Experimental

To a boiling methanol solution (50 ml) containing 2-acetylthiazole phenylthiosemicarbazone (1.38 g, 5 mmol) was added an equimolar of cadmium (II) iodide (1.38 g, 5 mmol) in 20 ml of methanol solution (Venkatraman *et al*., 2009)). The mixture was refluxed for 3 to 4 h under stirring. The resulting bright yellow solid obtained was filtered and dried (65% yield). Crystals suitable for diffraction were obtained from the mother liquor at ambient temperature after two days in a methanol–DMF mixture (5:1 V/V).

### Refinement

H atoms on C were placed in idealized positions with C—H distances 0.98 Å for methyl groups and 0.95 Å for others, and thereafter treated as riding. Coordinates of the NH hydrogen atoms were refined, with all N—H distances restrained to be equal. *U*_iso_ for H were assigned as 1.2 times *U*_eq_ of the attached atoms (1.5 for methyl).

### Crystal data

**Table:**

[Cd(C_12_H_11_N_4_S_2_)I(C_12_H_12_N_4_S_2_)]	*Z* = 2
*M**_r_* = 791.04	*F*(000) = 776
Triclinic, *P*1	*D*_x_ = 1.899 Mg m^−^^3^
Hall symbol: -P 1	Mo *K*α radiation, λ = 0.71073 Å
*a* = 8.6685 (4) Å	Cell parameters from 6604 reflections
*b* = 10.1323 (5) Å	θ = 2.5–30.9°
*c* = 16.7220 (8) Å	µ = 2.24 mm^−^^1^
α = 76.607 (2)°	*T* = 90 K
β = 79.481 (2)°	Needle, yellow
γ = 77.910 (2)°	0.13 × 0.06 × 0.05 mm
*V* = 1383.15 (11) Å^3^	

### Data collection

**Table:**

Bruker Kappa APEXII DUO CCD diffractometer	8142 independent reflections
Radiation source: fine-focus sealed tube	6715 reflections with *I* > 2σ(*I*)
TRIUMPH curved graphite monochromator	*R*_int_ = 0.024
φ and ω scans	θ_max_ = 31.0°, θ_min_ = 2.1°
Absorption correction: multi-scan (*SADABS*; Sheldrick, 2002)	*h* = −12→12
*T*_min_ = 0.760, *T*_max_ = 0.896	*k* = −14→14
14913 measured reflections	*l* = −23→23

### Refinement

**Table:**

Refinement on *F*^2^	Primary atom site location: structure-invariant direct methods
Least-squares matrix: full	Secondary atom site location: difference Fourier map
*R*[*F*^2^ > 2σ(*F*^2^)] = 0.027	Hydrogen site location: inferred from neighbouring sites
*wR*(*F*^2^) = 0.060	H atoms treated by a mixture of independent and constrained refinement
*S* = 1.02	*w* = 1/[σ^2^(*F*_o_^2^) + (0.0252*P*)^2^ + 0.1767*P*] where *P* = (*F*_o_^2^ + 2*F*_c_^2^)/3
8142 reflections	(Δ/σ)_max_ = 0.002
354 parameters	Δρ_max_ = 0.92 e Å^−^^3^
3 restraints	Δρ_min_ = −0.60 e Å^−^^3^

### Special details

**Table:**

Geometry. All e.s.d.'s (except the e.s.d. in the dihedral angle between two l.s. planes) are estimated using the full covariance matrix. The cell e.s.d.'s are taken into account individually in the estimation of e.s.d.'s in distances, angles and torsion angles; correlations between e.s.d.'s in cell parameters are only used when they are defined by crystal symmetry. An approximate (isotropic) treatment of cell e.s.d.'s is used for estimating e.s.d.'s involving l.s. planes.
Refinement. Refinement of *F*^2^ against ALL reflections. The weighted *R*-factor *wR* and goodness of fit *S* are based on *F*^2^, conventional *R*-factors *R* are based on *F*, with *F* set to zero for negative *F*^2^. The threshold expression of *F*^2^ > σ(*F*^2^) is used only for calculating *R*-factors(gt) *etc*. and is not relevant to the choice of reflections for refinement. *R*-factors based on *F*^2^ are statistically about twice as large as those based on *F*, and *R*- factors based on ALL data will be even larger.

### Fractional atomic coordinates and isotropic or equivalent isotropic displacement parameters (Å^2^)

**Table:**

	*x*	*y*	*z*	*U*_iso_*/*U*_eq_	
Cd1	0.330279 (19)	0.896637 (16)	0.264500 (10)	0.01247 (4)	
I1	0.213720 (18)	1.080081 (15)	0.128204 (9)	0.01602 (4)	
S1	0.89644 (7)	0.80381 (6)	0.20293 (4)	0.01885 (12)	
S2	0.16206 (7)	0.96342 (6)	0.39576 (4)	0.01535 (11)	
S3	0.98373 (7)	0.36995 (6)	0.41505 (4)	0.01949 (12)	
S4	0.27226 (7)	0.64947 (6)	0.26713 (4)	0.01495 (11)	
N1	0.5972 (2)	0.86012 (19)	0.19861 (12)	0.0142 (4)	
N2	0.5065 (2)	0.85083 (19)	0.36308 (12)	0.0128 (4)	
N3	0.4565 (2)	0.84714 (19)	0.44586 (12)	0.0140 (4)	
N4	0.2430 (2)	0.8941 (2)	0.54478 (12)	0.0133 (4)	
H4N	0.144 (2)	0.921 (3)	0.5524 (17)	0.016*	
N5	0.6877 (2)	0.4726 (2)	0.41015 (13)	0.0158 (4)	
N6	0.7257 (2)	0.49547 (19)	0.22678 (13)	0.0151 (4)	
N7	0.5761 (2)	0.5403 (2)	0.26598 (13)	0.0147 (4)	
H7N	0.570 (3)	0.534 (3)	0.3188 (13)	0.018*	
N8	0.4803 (2)	0.5489 (2)	0.14662 (13)	0.0174 (4)	
H8N	0.571 (3)	0.504 (3)	0.1322 (18)	0.021*	
C1	0.6688 (3)	0.8655 (2)	0.11853 (15)	0.0186 (5)	
H1	0.6100	0.8870	0.0731	0.022*	
C2	0.8309 (3)	0.8379 (3)	0.10863 (16)	0.0207 (5)	
H2	0.8978	0.8375	0.0569	0.025*	
C3	0.7028 (3)	0.8282 (2)	0.25083 (15)	0.0133 (4)	
C4	0.6581 (3)	0.8217 (2)	0.34029 (15)	0.0136 (4)	
C5	0.7822 (3)	0.7869 (2)	0.39688 (16)	0.0174 (5)	
H5A	0.7525	0.8454	0.4384	0.026*	
H5B	0.8853	0.8026	0.3644	0.026*	
H5C	0.7902	0.6899	0.4249	0.026*	
C6	0.3038 (3)	0.8948 (2)	0.46333 (14)	0.0136 (4)	
C7	0.3157 (3)	0.8592 (2)	0.61768 (14)	0.0137 (4)	
C8	0.4782 (3)	0.8135 (2)	0.62054 (15)	0.0175 (5)	
H8	0.5490	0.7996	0.5715	0.021*	
C9	0.5353 (3)	0.7884 (2)	0.69633 (16)	0.0208 (5)	
H9	0.6462	0.7581	0.6983	0.025*	
C10	0.4341 (3)	0.8066 (2)	0.76886 (16)	0.0201 (5)	
H10	0.4751	0.7900	0.8199	0.024*	
C11	0.2714 (3)	0.8496 (2)	0.76577 (15)	0.0175 (5)	
H11	0.2004	0.8610	0.8151	0.021*	
C12	0.2133 (3)	0.8755 (2)	0.69095 (15)	0.0154 (4)	
H12	0.1022	0.9049	0.6893	0.018*	
C13	0.7108 (3)	0.4442 (2)	0.49162 (15)	0.0181 (5)	
H13	0.6268	0.4618	0.5351	0.022*	
C14	0.8628 (3)	0.3890 (3)	0.50580 (16)	0.0200 (5)	
H14	0.8969	0.3644	0.5590	0.024*	
C15	0.8225 (3)	0.4389 (2)	0.36162 (15)	0.0152 (5)	
C16	0.8407 (3)	0.4521 (2)	0.27193 (15)	0.0151 (4)	
C17	1.0016 (3)	0.4115 (3)	0.22704 (16)	0.0172 (5)	
H17A	0.9970	0.4318	0.1672	0.026*	
H17B	1.0384	0.3126	0.2456	0.026*	
H17C	1.0758	0.4633	0.2387	0.026*	
C18	0.4532 (3)	0.5759 (2)	0.22228 (14)	0.0135 (4)	
C19	0.3685 (3)	0.5824 (2)	0.08851 (15)	0.0163 (5)	
C20	0.3172 (3)	0.7197 (2)	0.05317 (15)	0.0180 (5)	
H20	0.3514	0.7913	0.0696	0.022*	
C21	0.2159 (3)	0.7507 (3)	−0.00614 (15)	0.0186 (5)	
H21	0.1800	0.8441	−0.0305	0.022*	
C22	0.1664 (3)	0.6454 (3)	−0.03024 (15)	0.0186 (5)	
H22	0.0967	0.6669	−0.0710	0.022*	
C23	0.2185 (3)	0.5100 (3)	0.00503 (16)	0.0186 (5)	
H23	0.1848	0.4383	−0.0116	0.022*	
C24	0.3201 (3)	0.4775 (2)	0.06488 (15)	0.0166 (5)	
H24	0.3559	0.3841	0.0892	0.020*	

### Atomic displacement parameters (Å^2^)

**Table:**

	*U*^11^	*U*^22^	*U*^33^	*U*^12^	*U*^13^	*U*^23^
Cd1	0.01077 (8)	0.01618 (8)	0.01024 (8)	−0.00112 (6)	−0.00187 (6)	−0.00305 (6)
I1	0.01858 (8)	0.01534 (7)	0.01276 (8)	0.00038 (6)	−0.00397 (6)	−0.00170 (6)
S1	0.0111 (3)	0.0200 (3)	0.0233 (3)	−0.0016 (2)	0.0015 (2)	−0.0041 (2)
S2	0.0130 (3)	0.0212 (3)	0.0106 (3)	0.0018 (2)	−0.0028 (2)	−0.0042 (2)
S3	0.0147 (3)	0.0227 (3)	0.0211 (3)	−0.0013 (2)	−0.0068 (2)	−0.0030 (2)
S4	0.0135 (3)	0.0173 (3)	0.0147 (3)	−0.0032 (2)	−0.0017 (2)	−0.0042 (2)
N1	0.0121 (9)	0.0148 (9)	0.0151 (10)	−0.0030 (7)	−0.0001 (7)	−0.0022 (7)
N2	0.0131 (9)	0.0128 (9)	0.0126 (9)	−0.0025 (7)	−0.0012 (7)	−0.0030 (7)
N3	0.0142 (9)	0.0157 (9)	0.0125 (9)	−0.0031 (7)	−0.0036 (7)	−0.0015 (7)
N4	0.0121 (9)	0.0188 (9)	0.0099 (9)	−0.0030 (7)	−0.0021 (7)	−0.0039 (7)
N5	0.0152 (10)	0.0151 (9)	0.0185 (10)	−0.0040 (7)	−0.0035 (8)	−0.0038 (8)
N6	0.0140 (9)	0.0147 (9)	0.0182 (10)	−0.0036 (7)	−0.0006 (8)	−0.0067 (8)
N7	0.0130 (9)	0.0180 (9)	0.0136 (10)	−0.0012 (7)	−0.0025 (8)	−0.0048 (8)
N8	0.0161 (10)	0.0201 (10)	0.0163 (10)	0.0027 (8)	−0.0053 (8)	−0.0072 (8)
C1	0.0194 (12)	0.0217 (12)	0.0143 (11)	−0.0045 (9)	0.0015 (9)	−0.0051 (9)
C2	0.0192 (12)	0.0238 (12)	0.0169 (12)	−0.0030 (10)	0.0041 (10)	−0.0053 (10)
C3	0.0104 (10)	0.0115 (10)	0.0182 (11)	−0.0035 (8)	0.0006 (9)	−0.0043 (9)
C4	0.0138 (11)	0.0101 (10)	0.0165 (11)	−0.0032 (8)	−0.0031 (9)	−0.0004 (8)
C5	0.0148 (11)	0.0185 (11)	0.0193 (12)	−0.0016 (9)	−0.0073 (9)	−0.0020 (9)
C6	0.0162 (11)	0.0143 (10)	0.0116 (10)	−0.0038 (8)	−0.0033 (9)	−0.0032 (8)
C7	0.0197 (11)	0.0106 (10)	0.0125 (11)	−0.0054 (8)	−0.0055 (9)	−0.0007 (8)
C8	0.0193 (12)	0.0186 (11)	0.0152 (11)	−0.0031 (9)	−0.0050 (9)	−0.0029 (9)
C9	0.0219 (13)	0.0197 (12)	0.0220 (13)	−0.0048 (10)	−0.0090 (10)	−0.0013 (10)
C10	0.0302 (14)	0.0158 (11)	0.0173 (12)	−0.0049 (10)	−0.0125 (10)	−0.0017 (9)
C11	0.0283 (13)	0.0129 (10)	0.0114 (11)	−0.0042 (9)	−0.0051 (10)	−0.0004 (9)
C12	0.0187 (12)	0.0130 (10)	0.0152 (11)	−0.0038 (9)	−0.0041 (9)	−0.0021 (9)
C13	0.0195 (12)	0.0203 (12)	0.0151 (12)	−0.0052 (9)	−0.0039 (9)	−0.0018 (9)
C14	0.0218 (13)	0.0222 (12)	0.0169 (12)	−0.0057 (10)	−0.0073 (10)	−0.0006 (10)
C15	0.0174 (11)	0.0111 (10)	0.0189 (12)	−0.0035 (8)	−0.0071 (9)	−0.0025 (9)
C16	0.0171 (11)	0.0125 (10)	0.0171 (12)	−0.0033 (8)	−0.0044 (9)	−0.0036 (9)
C17	0.0115 (11)	0.0228 (12)	0.0192 (12)	−0.0024 (9)	−0.0010 (9)	−0.0094 (10)
C18	0.0131 (10)	0.0132 (10)	0.0139 (11)	−0.0034 (8)	−0.0017 (8)	−0.0015 (8)
C19	0.0132 (11)	0.0214 (12)	0.0140 (11)	0.0000 (9)	−0.0042 (9)	−0.0039 (9)
C20	0.0197 (12)	0.0182 (11)	0.0174 (12)	−0.0034 (9)	−0.0033 (10)	−0.0055 (9)
C21	0.0207 (12)	0.0183 (11)	0.0144 (11)	−0.0007 (9)	−0.0015 (9)	−0.0014 (9)
C22	0.0146 (11)	0.0285 (13)	0.0128 (11)	−0.0033 (9)	−0.0038 (9)	−0.0035 (10)
C23	0.0200 (12)	0.0213 (12)	0.0170 (12)	−0.0070 (10)	−0.0010 (10)	−0.0070 (10)
C24	0.0176 (12)	0.0137 (10)	0.0175 (12)	−0.0028 (9)	−0.0022 (9)	−0.0012 (9)

### Geometric parameters (Å, º)

**Table:**

Cd1—N2	2.3524 (19)	C5—H5A	0.9800
Cd1—N1	2.3673 (19)	C5—H5B	0.9800
Cd1—S2	2.5510 (6)	C5—H5C	0.9800
Cd1—S4	2.6449 (6)	C7—C8	1.393 (3)
Cd1—I1	2.7860 (2)	C7—C12	1.397 (3)
S1—C2	1.709 (3)	C8—C9	1.394 (3)
S1—C3	1.716 (2)	C8—H8	0.9500
S2—C6	1.748 (2)	C9—C10	1.386 (4)
S3—C14	1.706 (3)	C9—H9	0.9500
S3—C15	1.731 (2)	C10—C11	1.393 (4)
S4—C18	1.708 (2)	C10—H10	0.9500
N1—C3	1.320 (3)	C11—C12	1.382 (3)
N1—C1	1.363 (3)	C11—H11	0.9500
N2—C4	1.291 (3)	C12—H12	0.9500
N2—N3	1.367 (3)	C13—C14	1.360 (3)
N3—C6	1.314 (3)	C13—H13	0.9500
N4—C6	1.367 (3)	C14—H14	0.9500
N4—C7	1.413 (3)	C15—C16	1.457 (3)
N4—H4N	0.842 (19)	C16—C17	1.483 (3)
N5—C15	1.325 (3)	C17—H17A	0.9800
N5—C13	1.368 (3)	C17—H17B	0.9800
N6—C16	1.302 (3)	C17—H17C	0.9800
N6—N7	1.378 (3)	C19—C24	1.379 (3)
N7—C18	1.342 (3)	C19—C20	1.392 (3)
N7—H7N	0.864 (19)	C20—C21	1.382 (3)
N8—C18	1.325 (3)	C20—H20	0.9500
N8—C19	1.432 (3)	C21—C22	1.393 (3)
N8—H8N	0.85 (2)	C21—H21	0.9500
C1—C2	1.360 (3)	C22—C23	1.377 (3)
C1—H1	0.9500	C22—H22	0.9500
C2—H2	0.9500	C23—C24	1.391 (3)
C3—C4	1.465 (3)	C23—H23	0.9500
C4—C5	1.495 (3)	C24—H24	0.9500
			
N2—Cd1—N1	69.91 (7)	C12—C7—N4	115.6 (2)
N2—Cd1—S2	74.23 (5)	C7—C8—C9	119.1 (2)
N1—Cd1—S2	141.74 (5)	C7—C8—H8	120.5
N2—Cd1—S4	102.87 (5)	C9—C8—H8	120.5
N1—Cd1—S4	96.47 (5)	C10—C9—C8	121.5 (2)
S2—Cd1—S4	104.16 (2)	C10—C9—H9	119.2
N2—Cd1—I1	146.34 (5)	C8—C9—H9	119.2
N1—Cd1—I1	94.99 (5)	C9—C10—C11	119.0 (2)
S2—Cd1—I1	107.969 (15)	C9—C10—H10	120.5
S4—Cd1—I1	108.767 (14)	C11—C10—H10	120.5
C2—S1—C3	90.02 (12)	C12—C11—C10	120.0 (2)
C6—S2—Cd1	97.99 (8)	C12—C11—H11	120.0
C14—S3—C15	89.54 (12)	C10—C11—H11	120.0
C18—S4—Cd1	100.32 (8)	C11—C12—C7	120.9 (2)
C3—N1—C1	111.6 (2)	C11—C12—H12	119.6
C3—N1—Cd1	113.40 (15)	C7—C12—H12	119.6
C1—N1—Cd1	134.96 (16)	C14—C13—N5	115.1 (2)
C4—N2—N3	116.89 (19)	C14—C13—H13	122.5
C4—N2—Cd1	119.85 (16)	N5—C13—H13	122.5
N3—N2—Cd1	123.19 (14)	C13—C14—S3	110.70 (19)
C6—N3—N2	113.65 (19)	C13—C14—H14	124.7
C6—N4—C7	132.1 (2)	S3—C14—H14	124.7
C6—N4—H4N	113.3 (19)	N5—C15—C16	125.7 (2)
C7—N4—H4N	114.6 (19)	N5—C15—S3	113.64 (18)
C15—N5—C13	111.0 (2)	C16—C15—S3	120.61 (18)
C16—N6—N7	117.6 (2)	N6—C16—C15	125.3 (2)
C18—N7—N6	118.8 (2)	N6—C16—C17	116.1 (2)
C18—N7—H7N	126.0 (19)	C15—C16—C17	118.5 (2)
N6—N7—H7N	114.9 (19)	C16—C17—H17A	109.5
C18—N8—C19	126.4 (2)	C16—C17—H17B	109.5
C18—N8—H8N	117 (2)	H17A—C17—H17B	109.5
C19—N8—H8N	117 (2)	C16—C17—H17C	109.5
C2—C1—N1	114.9 (2)	H17A—C17—H17C	109.5
C2—C1—H1	122.6	H17B—C17—H17C	109.5
N1—C1—H1	122.6	N8—C18—N7	117.1 (2)
C1—C2—S1	110.05 (19)	N8—C18—S4	124.02 (18)
C1—C2—H2	125.0	N7—C18—S4	118.89 (18)
S1—C2—H2	125.0	C24—C19—C20	120.9 (2)
N1—C3—C4	122.9 (2)	C24—C19—N8	119.3 (2)
N1—C3—S1	113.40 (18)	C20—C19—N8	119.7 (2)
C4—C3—S1	123.67 (17)	C21—C20—C19	119.3 (2)
N2—C4—C3	113.9 (2)	C21—C20—H20	120.4
N2—C4—C5	125.2 (2)	C19—C20—H20	120.4
C3—C4—C5	120.9 (2)	C20—C21—C22	120.2 (2)
C4—C5—H5A	109.5	C20—C21—H21	119.9
C4—C5—H5B	109.5	C22—C21—H21	119.9
H5A—C5—H5B	109.5	C23—C22—C21	119.9 (2)
C4—C5—H5C	109.5	C23—C22—H22	120.0
H5A—C5—H5C	109.5	C21—C22—H22	120.0
H5B—C5—H5C	109.5	C22—C23—C24	120.4 (2)
N3—C6—N4	117.7 (2)	C22—C23—H23	119.8
N3—C6—S2	128.92 (18)	C24—C23—H23	119.8
N4—C6—S2	113.34 (17)	C19—C24—C23	119.3 (2)
C8—C7—C12	119.4 (2)	C19—C24—H24	120.3
C8—C7—N4	125.0 (2)	C23—C24—H24	120.3
			
N2—Cd1—S2—C6	−9.65 (9)	N2—N3—C6—N4	179.49 (18)
N1—Cd1—S2—C6	−30.51 (11)	N2—N3—C6—S2	−1.4 (3)
S4—Cd1—S2—C6	89.99 (8)	C7—N4—C6—N3	5.1 (4)
I1—Cd1—S2—C6	−154.51 (7)	C7—N4—C6—S2	−174.19 (19)
N2—Cd1—S4—C18	−69.76 (10)	Cd1—S2—C6—N3	10.5 (2)
N1—Cd1—S4—C18	1.05 (10)	Cd1—S2—C6—N4	−170.29 (15)
S2—Cd1—S4—C18	−146.47 (8)	C6—N4—C7—C8	−0.7 (4)
I1—Cd1—S4—C18	98.58 (8)	C6—N4—C7—C12	178.1 (2)
N2—Cd1—N1—C3	2.61 (15)	C12—C7—C8—C9	−1.6 (3)
S2—Cd1—N1—C3	24.0 (2)	N4—C7—C8—C9	177.2 (2)
S4—Cd1—N1—C3	−98.76 (15)	C7—C8—C9—C10	0.7 (4)
I1—Cd1—N1—C3	151.67 (15)	C8—C9—C10—C11	0.7 (4)
N2—Cd1—N1—C1	−176.6 (2)	C9—C10—C11—C12	−1.0 (3)
S2—Cd1—N1—C1	−155.22 (18)	C10—C11—C12—C7	0.0 (3)
S4—Cd1—N1—C1	82.0 (2)	C8—C7—C12—C11	1.3 (3)
I1—Cd1—N1—C1	−27.6 (2)	N4—C7—C12—C11	−177.6 (2)
N1—Cd1—N2—C4	−3.14 (16)	C15—N5—C13—C14	0.1 (3)
S2—Cd1—N2—C4	−169.57 (17)	N5—C13—C14—S3	−0.3 (3)
S4—Cd1—N2—C4	89.12 (16)	C15—S3—C14—C13	0.33 (19)
I1—Cd1—N2—C4	−70.69 (19)	C13—N5—C15—C16	178.5 (2)
N1—Cd1—N2—N3	179.88 (17)	C13—N5—C15—S3	0.2 (2)
S2—Cd1—N2—N3	13.46 (14)	C14—S3—C15—N5	−0.31 (18)
S4—Cd1—N2—N3	−87.86 (15)	C14—S3—C15—C16	−178.66 (19)
I1—Cd1—N2—N3	112.34 (15)	N7—N6—C16—C15	4.1 (3)
C4—N2—N3—C6	171.65 (19)	N7—N6—C16—C17	−176.95 (18)
Cd1—N2—N3—C6	−11.3 (2)	N5—C15—C16—N6	−2.1 (4)
C16—N6—N7—C18	−175.2 (2)	S3—C15—C16—N6	176.07 (18)
C3—N1—C1—C2	−0.1 (3)	N5—C15—C16—C17	179.0 (2)
Cd1—N1—C1—C2	179.13 (16)	S3—C15—C16—C17	−2.9 (3)
N1—C1—C2—S1	0.0 (3)	C19—N8—C18—N7	−177.6 (2)
C3—S1—C2—C1	0.08 (19)	C19—N8—C18—S4	3.7 (3)
C1—N1—C3—C4	177.1 (2)	N6—N7—C18—N8	8.5 (3)
Cd1—N1—C3—C4	−2.3 (3)	N6—N7—C18—S4	−172.77 (15)
C1—N1—C3—S1	0.2 (2)	Cd1—S4—C18—N8	−108.70 (19)
Cd1—N1—C3—S1	−179.24 (9)	Cd1—S4—C18—N7	72.63 (18)
C2—S1—C3—N1	−0.15 (18)	C18—N8—C19—C24	−115.4 (3)
C2—S1—C3—C4	−177.1 (2)	C18—N8—C19—C20	68.2 (3)
N3—N2—C4—C3	−179.80 (18)	C24—C19—C20—C21	0.3 (4)
Cd1—N2—C4—C3	3.0 (2)	N8—C19—C20—C21	176.6 (2)
N3—N2—C4—C5	−1.1 (3)	C19—C20—C21—C22	−0.2 (4)
Cd1—N2—C4—C5	−178.23 (16)	C20—C21—C22—C23	−0.1 (4)
N1—C3—C4—N2	−0.4 (3)	C21—C22—C23—C24	0.2 (4)
S1—C3—C4—N2	176.24 (16)	C20—C19—C24—C23	−0.1 (4)
N1—C3—C4—C5	−179.2 (2)	N8—C19—C24—C23	−176.4 (2)
S1—C3—C4—C5	−2.6 (3)	C22—C23—C24—C19	−0.1 (4)

### Hydrogen-bond geometry (Å, º)

**Table:**

*D*—H···*A*	*D*—H	H···*A*	*D*···*A*	*D*—H···*A*
N4—H4*N*···S2^i^	0.84 (2)	2.74 (2)	3.559 (2)	166 (2)
N7—H7*N*···N5	0.86 (2)	1.90 (2)	2.651 (3)	144 (3)

Symmetry code: (i) −*x*, −*y*+2, −*z*+1.
